# Overexpression of a SBP-Box Gene (*VpSBP16*) from Chinese Wild *Vitis* Species in *Arabidopsis* Improves Salinity and Drought Stress Tolerance

**DOI:** 10.3390/ijms19040940

**Published:** 2018-03-22

**Authors:** Hongmin Hou, Hui Jia, Qin Yan, Xiping Wang

**Affiliations:** 1 State Key Laboratory of Crop Stress Biology in Arid Areas, College of Horticulture, Northwest A&F University, Yangling 712100, China; hmhou@qau.edu.cn (H.H.); jhhsunshine@nwafu.edu.cn (H.J.); yanqin0421@gmail.com (Q.Y.); 2 Key Laboratory of Horticultural Crop Biology and Germplasm Innovation in Northwest China, Ministry of Agriculture, Yangling 712100, China; 3 College of Horticulture, Qingdao Agricultural University, Qingdao 266109, China; 4 Qingdao Key Laboratory of Genetic Development and Breeding in Horticultural Plants, Qingdao Agricultural University, Qingdao 266109, China

**Keywords:** *Vitis pseudoreticulata*, *VpSBP16*, salt, drought, ROS, SOS

## Abstract

Salinity and drought are two major abiotic stresses that limit grape productivity. Responses to stress in grape are known to be regulated by several families of transcription factors. However, little is known about the role of grape *Squamosa* promoter binding protein (SBP)-box transcription factor genes in response to abiotic stress. To better understand the functions of the grape SBP-box genes in abiotic stress tolerance, a full-length complementary DNA (cDNA) sequence of the putative SBP-box transcription factor gene, *VpSBP16* was amplified from Chinese wild grapevine *Vitis pseudoreticulata* clone “Baihe-35-1”. We observed that the VpSBP16 protein fused to the green fluorescent protein (GFP) reporter accumulated in the nucleus when transiently expressed in onion epidermal cells. Moreover, VpSBP16 was shown to have transcriptional activation activity using a yeast *trans*-activation assay. We performed a *VpSBP16* functional analysis through the characterization of transgenic *Arabidopsis thaliana* plants constitutively over-expressing *VpSBP16.* The transgenic lines had longer roots and the seeds had a higher germination rate than the wild type (WT) under osmotic stress. In addition, the accumulation of reactive oxygen species (ROS) of transgenic seedlings was significantly lower than WT in the transgenic lines, as was electrolyte leakage. *VpSBP16* overexpression also elevated expression levels of stress-response genes involved in the salt overly sensitive (SOS) pathway. These results indicate that overexpression *VpSBP16* in *A*. *thaliana* enhances tolerance of salt and drought stress during seed germination, as well in seedlings and mature plants, by regulating SOS and ROS signaling cascades.

## 1. Introduction

Plants frequently encounter stressful environmental conditions, such as extreme temperatures, drought, and high salinity, which can severely limit growth and development and greatly reduce the quality and yield of crops. To prevent the potentially harmful effects of such stresses, plants have evolved a range of physiological, biochemical, molecular, and cellular processes that represent tolerance or avoidance mechanisms.

Transcriptional control of the expression of stress-responsive genes is a crucial part of plant responses to abiotic stress and many transcription factors have been identified that regulate responses to environmental cues by activating or repressing multiple target genes. For example, *MYB2*, *MYB15* [1,2]; *bZIP24* [3]; *WRKY25*, *WRKY33*, *WRKY63/ABO3*, *WRKY18*, *WRKY60* [4,5,6] from *Arabidopsis thaliana*, *OsMPS* [7] from rice (*Oryza sativa*) and *SlAREB* [8] from tomato (*Solanum lycopersicum*) have all been reported to regulate responses to some abiotic stresses by activating or repressing transcription of multiple target genes, such as *RD29A*, *COR15*, *KIN1*.

Squamosa promoter binding protein (SBP)-box genes encode a family of transcription factors that are exclusively present in plants [9,10,11]. A common feature of SBP-box genes is that the corresponding proteins contain a highly conserved SBP-domain, an assembly of approximately 76 amino acid residues that includes two zinc fingers and a nuclear localization signal [9,12,13]. To date, several important and divergent biological processes have been reported to be regulated by SBP-box genes. However, only a few SBP genes have been shown to play a role in responses to abiotic and biotic stresses. For example, SBP genes in Arabidopsis are responsive to various biotic and abiotic stress, and interact with genes involved in the defense response pathway [14]. *Arabidopsis AtSPL14* has been associated with programmed cell death and to play a role in sensitivity to the fungal toxin fumonisin B1 [15]. The birch *BpSPL9* gene was reported to increase resistance to abiotic stress by enhancing the activities of superoxide dismutase (SOD) and peroxidase (POD) in transgenic lines [16]. Furthermore, preliminary expression profiling of 31 maize *ZmSPL* genes showed that some genes were influenced by several environmental stimuli, including drought, cold, salinity, and abscisic acid exposure [17]. It has also been reported that responses to heat stress mediated by the micro-RNA, miR156, operate through SPL transcription factors (*SPL2*, *SPL9* and *SPL11*) in *A. thaliana* [18]. Overexpression of *OsmiR156k* in rice was reported to reduce tolerance to cold stress by down-regulating *SPL3*, *SPL14* and *SPL17* [19], while *SPL9*, *SPL10* and *SOC1* from *A. thaliana* were downregulated under stress conditions and upregulated after recovery from stress conditions [20]. Lastly, miR156 was shown to improve drought tolerance in alfalfa, at least in part by silencing *SPL13* [21].

In contrast to the extensive studies in model species such as *A. thaliana*, relatively little is known about SBP-box genes from grape. We previously found that *VvSBP17*, the ortholog of *A. thaliana AtSPL14*, was up-regulated after infection with the phytoplasma Bois Noir in the susceptible *V. vinifera* cultivar ‘Chardonnay’ [22]. Moreover, it has been suggested that *VpSBP5* from *V. pseudoreticulata* participates in the regulation of resistance to *Erisyphe necator* by inducing salicylic acid (SA) and methyl jasmonate (MeJA) molecular signals [23]. However, a role for SBP-box genes in responses to abiotic stresses in grape has yet to be demonstrated. In the present study, we describe the functional evaluation of an SBP gene, *VpSBP16*, from a *Vitis* wild species following its over expression in *A. thaliana*, and present data that support a role of its role enhancing abiotic stress resistance.

## 2. Results

### 2.1. Cloning and Sequence Analysis of VpSBP16

**Figure 1:**
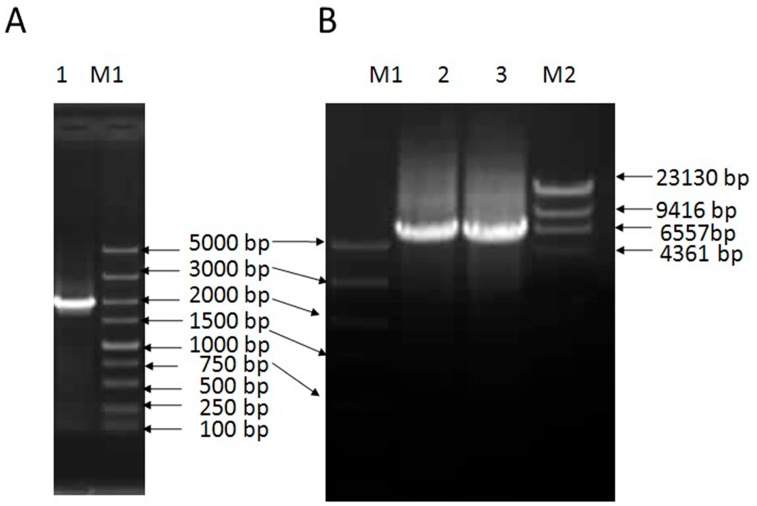
Cloning of *VpSBP16* from *V*. *pesudoreticulata*. (**A**) PCR amplification of the full-length *VpSBP16* cDNA and (**B**) DNA from *Vitis pseudoreticulata*. M1: DNA marker DL5000; M2: DNA marker λ-Hind III; 1: *VpSBP16* PCR product from cDNA; 2 and 3: *VpSBP16* PCR product.

**Figure 2:**
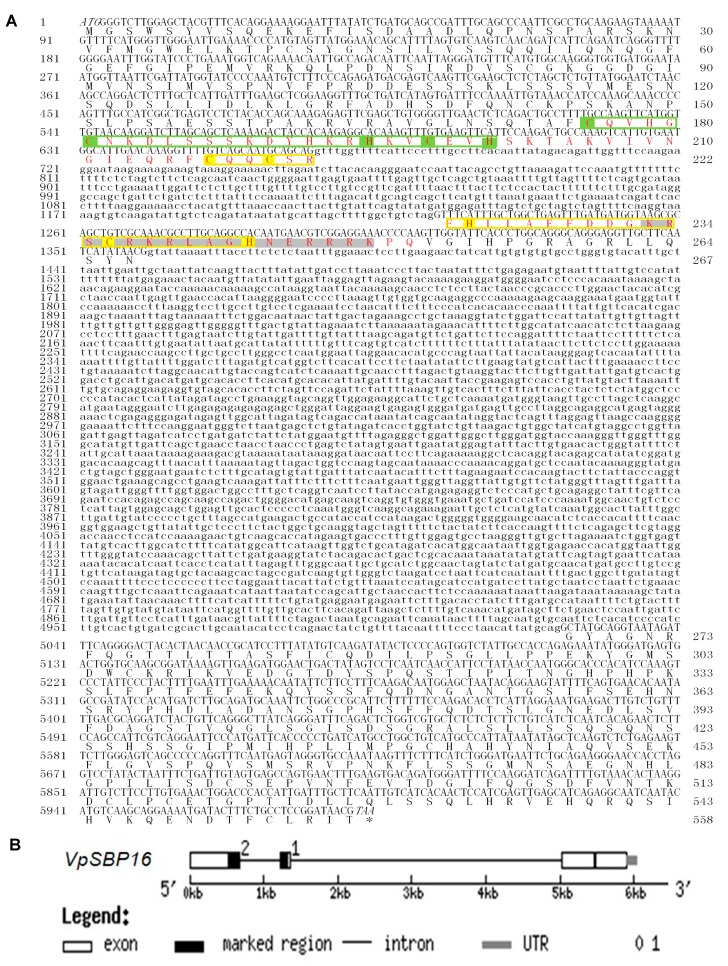
The DNA, cDNA nucleotide sequence and deduced amino acid sequence of *VpSBP16* from *V*. *pesudoreticulata* (**A**) and Exon-intron structures of *VpSBP16* (**B**). The SBP domain is shown in red and the two zinc-binding sites of the C2HCH type (zinc finger 1 and zinc finger 2) are indicated with green and yellow boxes. The conserved basic amino acids of the nuclear location signal are shaded in dark grey.

A 5984 base pair (bp) full-length *VpSBP16* DNA sequence, including a 1647 bp open reading frame (ORF), was amplified from complementary DNA (cDNA) (Figure 1A) or genomic DNA (Figure 1B) extracted from the leaves of *V. pseudoreticulata* W. T. Wang clone ‘Baihe-35-1’, using the primers based on the cDNA sequence of *VvSBP16* obtained from the Grape Genome Database (http://www.genoscope.cns.fr). A comparison between the genomic DNA and cDNA sequence revealed that the coding region of *VpSBP16* has two introns (557 bp and 3662 bp), the relative positions of which are shown in Figure 2. The predicted VpSBP16 protein contains a highly conserved SBP-domain with two zinc-binding sites of the C2HCH type (zinc finger 1 and zinc finger 2), together with a nuclear localization signal (NLS) (Figure 2A).

### 2.2. Subcellular Localization and Function of VpSBP16 in Transcriptional Activation

**Figure 3:**
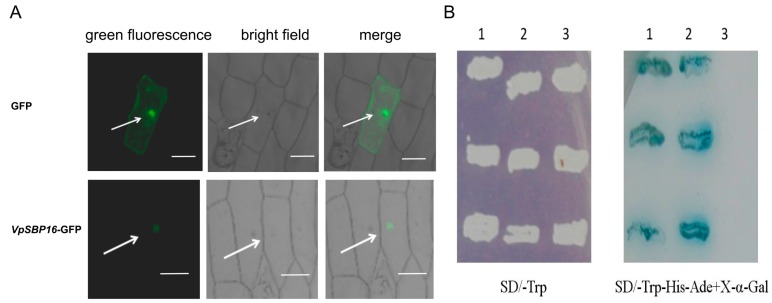
Subcellular localization and transcriptional activation function of VpSBP16. (**A**) Subcellular localization of the VpSBP16-GFP (bottom row) fusion protein (top row) in onion epidermal cells. White arrowheads indicate the location of the nucleus in onion epidermal cell, scale bars: 50 μm. (**B**) Transcriptional activation function of VpSBP16 in yeast. Yeast cells containing the different plasmids grown on SD/-Trp select medium (**left**). Yeast cells containing the different plasmids grown on SD/-Trp-His-Ade+X-α-gal selection medium (**right**). 1: Positive control. (pGBKT7-Gal4); 2: pGBKT7-VpSBP16; 3: Negative control (pGBKT7). The experiments were repeated three times with consistent results.

Sequence analysis of the grape SBP-box genes revealed that their deduced protein sequences contained putative NLS regions (Figure 2A). To confirm targeting of *VpSBP16* to the nucleus, the VpSBP16 coding sequence (CDS) without the C terminal termination codon was translationally fused to green fluorescent protein (GFP) coding sequence in the pBI221-GFP vector to generate the pBI221-*VpSBP16*-GFP vector. This vector and a pBI221-GFP negative control vector were transformed into onion epidermal cells using particle bombardment [24] for transient expression analysis. The VpSBP16-GFP fusion protein was observed by confocal microscopic analysis to accumulate in the nucleus of onion epidermal cells, whereas the GFP control protein was distributed throughout the cell (Figure 3A), consistent with the prediction that VpSBP16 is a nuclear protein that functions as a transcription factor.

To investigate whether VpSBP16 has transcriptional activation activity, the full-length VpSBP16 CDS and the yeast *GAL4* cDNA were separately fused to the *GAL4* DNA-binding domain in the pGBKT7 vector. Yeast cells transformed with the pGBKT7 control vector only grew on Single Dropout (-Trp) (SD/-Trp) medium, while those transformed with the *VpSBP16* and *GAL4* plasmids were able to grow on both SD/-Trp and SD/-Trp/-Ade/-His media, and exhibited blue staining in X-α-gal solution (Figure 3B), demonstrating that the VpSBP16 protein can function as a transcriptional activator.

### 2.3. Effect of Osmotic Stress on Seed Germination in Transgenic VpSBP16 Arabidopsis Lines

**Figure 4:**
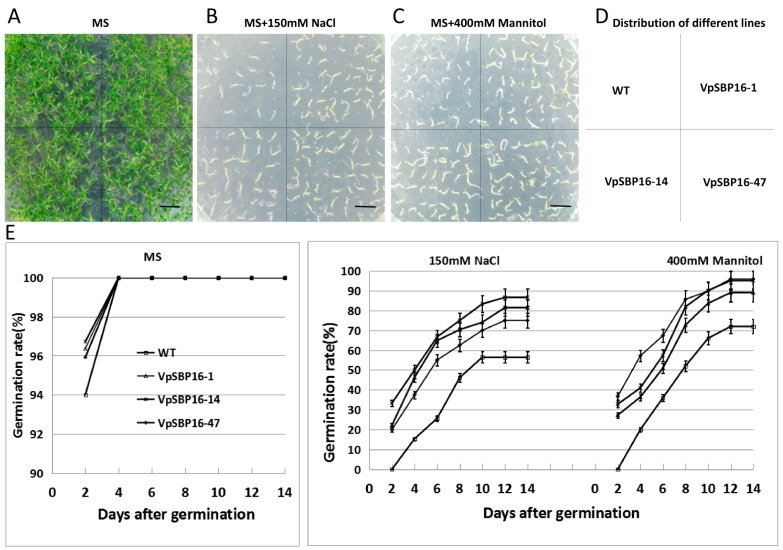
Phenotypes of wild type (WT) and *VpSBP16* transgenic *Arabidopsis thaliana* lines at the seed germination stage placed under osmotic stress (**A**–**D**) Photographs of seed germination in WT and transgenic lines 14 days after seeds were cultivated on Murashige-Skoog (MS) basal medium, MS basal medium supplemented with 150 mM NaCl or 400 mM mannitol. The experiments were repeated three times with consistent results, scale bars: 1 cm. (**E**) Seed germination rates of WT and transgenic lines cultivated on MS basal medium, MS basal medium supplemented with 150 mM NaCl or 400 mM mannitol for 14 days, respectively. Each data point is the mean of three replicates of 100–150 seeds. The error bars indicate the SD.

**Figure 5:**
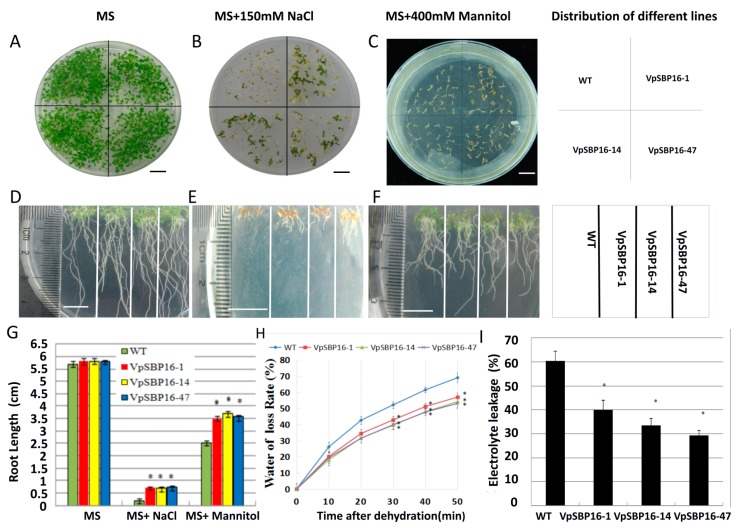
Analysis of the osmotic tolerance of WT and *VpSBP16* transgenic *A. thaliana* seedlings. (**A**–**C**) Photographs of seedlings sown on MS basal medium, MS basal medium supplemented with 150 mM NaCl or 400 mM mannitol for 14, 35 and 20 days, respectively. The experiments were repeated three times with consistent results, scale bars: 1 cm. (**D**–**G**) Root growth (**D**–**F**) and root length (**G**) of WT and transgenic lines grown on MS basal medium, MS basal medium supplemented with 150 mM NaCl or 400 mM mannitol for 14, 35 and 20days, respectively. The experiments were repeated three times with consistent results, scale bars: 1 cm. (**H**) Water loss rate from 2-week-old detached transgenic and WT plants grown on the same MS basal medium, measured over a 50 min experimentation period. (**I**) Electrolyte leakage of seedlings of WT and the VpSBP16-1, VpSBP16-14 and VpSBP16-47 transgenic plants sampled at the last time point of dehydration. Asterisks indicated values that are significantly different from WT (*n* = 50 for each genotype, * 0.01< *p* < 0.05, one-way ANOVA). Each data point is the mean of three replicates of ten detached plants.

We generated transgenic *A. thaliana* plants expressing the *VpSBP16* ORF under the regulation of the constitutive CaMV 35S promoter to determine whether the gene confers abiotic stress tolerance. A total of 89 independent transgenic lines were obtained and the presence of the transgene confirmed by PCR and kanamycin antibiotic selection. The expression level of *VpSBP16* in twelve transgenic lines with the better performance under salt stress was verified by semi-quantitative RT-PCR with transcript-specific primers *VpSBP16*-F and *VpSBP16*-R (Table 1).The three lines (VpSBP16-1, VpSBP16-14, and VpSBP16-47) with the best performance and the highest levels of expression were selected for further study (Figure S1), and grown in the same Petri dish or pot with different stress conditions as WT plants (Figure 4 and Figure 5).

We measured the germination rates of seeds from the three transgenic Arabidopsis lines and WT plants placed on Murashige and Skoog (MS) medium, MS medium with 150 mM NaCl and MS medium with 400 mM mannitol (Figure 4). Two days after sowing, seeds from the three transgenic lines (VpSBP16-1, VpSBP16-14 and VpSBP16-47) and wild type (WT) began to germinate on MS medium, while at 4 days after germination the rate nearly was 100% (Figure 4E). The germination rate of the transgenic lines (VpSBP16-1, VpSBP16-14 and VpSBP16-47) on 150 mM NaCl medium was 81.5%, 86.7% and 75.0% respectively, 14 days after culturing. However, the germination rate of wild type was only 56.4%. On the MS medium with 400 mM mannitol, the result was similar, with the WT showing a lower germination rate (Figure 4E). In general, compared to MS medium, the germination of all seeds on the MS medium with 150 mM NaCl or 400 mM mannitol was lower, while the transgenic seeds exhibited 17–32% higher germination rates than WT seeds sown on the same media (Figure 4E).

### 2.4. VpSBP16 Transgenic Arabidopsis Seedling Resistance to Osmotic Stress

To assess whether *VpSBP16* confers resistance to osmotic stress, the seedling of three transgenic lines (VpSBP16-1, VpSBP16-14 and VpSBP16-47) and WT were grown on MS medium containing 150 mM NaCl or 400 mM mannitol. Both the WT and transgenic seedling displayed similar growth characteristics on MS basal medium (Figure 5A). However, upon exposure to 150 mM NaCl, their growth was strikingly different after 35 days. Three transgenic lines had not only a higher seed germination rate, but also larger root systems and leaves that were greener (Figure 5B,E). The WT seedlings had withered yellow leaves, grew slower and did not form normal cotyledons (Figure 5B). Moreover, the root lengths of the WT seedlings were 0.15–0.2 cm, while those of the transgenic seedlings were approximately 4-fold greater (Figure 5D,E,G), indicating that overexpression of the *VpSBP16* gene offsets some of the inhibitory effects of salt stress on root elongation. The seedlings grown on medium with 400 mM mannitol had the same growth characteristics as those grown under salt stress (Figure 5C,F,G).

We also measured the transpirational water loss of seedlings from 10-day-old WT and transgenic plants left on the laboratory bench at room temperature with a humidity of 40–45%, every 10 min over a 50 min period. Less water loss was detected in the three transgenic lines than in the WT control (Figure 5H). We next measured changes in relative electrolyte leakage in the transgenic and WT seedlings to determine whether there was a correlation with the improved osmotic stress tolerance of the *VpSBP16* transgenic seedlings. The relative electrolyte leakage was also significantly lower in transgenic lines than in WT (Figure 5I). These results suggest that osmotic damage in the transgenic lines was significantly lower than in the control and overexpression of *VpSBP16* significantly improved resistance to osmotic stress.

### 2.5. Response to Abiotic Stresses of VpSBP16 Transgenic Arabidopsis Plants

**Figure 6:**
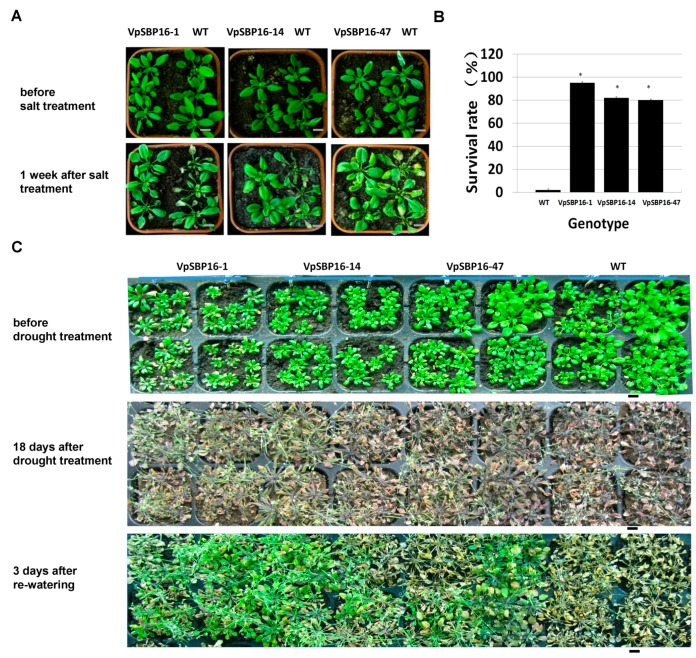
Growth of WT and *VpSBP16* transgenic *A. thaliana* plants in pots under non-stress or osmotic stress conditions. (**A**) Representative photographs of 3-week-old WT and transgenic lines before salt treatment and after salt treatment for 1 week. The experiments were repeated three times with consistent results, scale bars: 1 cm.(**B**) Survival rates of WT and transgenic lines 3 days after re-watering. Each data point is the mean of three replicates. The error bars indicate the SD. (*n* = 50 for each genotype, * 0.01< *p* < 0.05, one-way ANOVA). (**C**) Representative photographs showing the phenotype of plants before drought, 18 days after drought treatment and 3 days after re-watering. The experiments were repeated three times with consistent results, scale bars: 1 cm.

The results described above indicated that overexpression of *VpSBP16* involved resistance of seedlings to salt stress and short-term drought stress. We next examined the responses of adult plants. Three-week-old transgenic and WT plants grown in the same pot were irrigated with 300 mM NaCl solution. Most leaves of the WT plants showed etiolated and wilted symptoms after 1 week of treatment. In contrast, those of the transgenic plants remained green or showed only mild etiolation under the same treatments (Figure 6A). The growth and development of 2-week-old transgenic lines and WT grown in the same pot following the drought treatment for 18 days was then investigated. All WT and transgenic plants obviously withered and exhibited severe water loss related symptoms, and after rewatering for 3 days, most of transgenic plants had resumed normal growth (73–80% recovery rate), but almost all the WT plants had died (2% recovery rate, Figure 6B,C).

**Figure 7:**
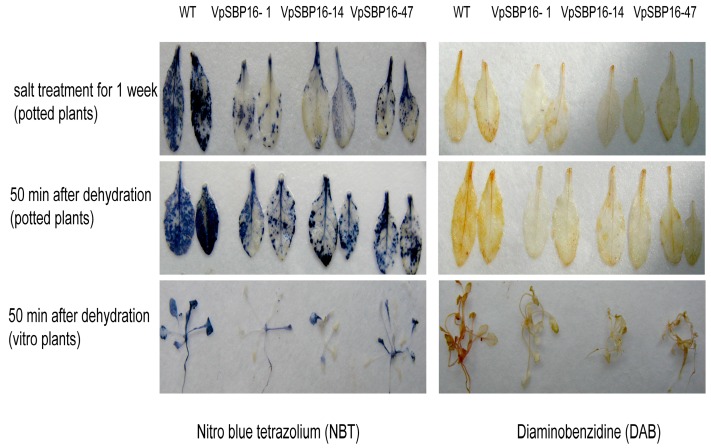
Histochemical staining assay of ROS accumulation with nitro blue tetrazolium (NBT) and diaminobenzidine (DAB) in 2-week-old wild type (WT), or VpSBP16-1, VpSBP16-14 and VpSBP16-47 transgenic lines after salt treatment for 1 week or after the transpirational water loss from 50 min. The experiment was repeated three times with 5–10 leaves or plants.

Substantial accumulation of reactive oxygen species (ROS) because of exposure to stress can cause progressive oxidative damage, ultimately leading to cell injury and even death [25]. We measured levels of the ROS species O_2_^−^ and H_2_O_2_ in leaves of plants grown under water loss and salt stress by NBT and DAB histochemical staining, respectively. We observed that leaves of the transgenic lines accumulated much lower levels of O_2_^−^ or H_2_O_2_ than those of WT plants (Figure 7).

### 2.6. Altered Expression of Abiotic Stress Responsive Genes in Transgenic VpSBP16 Plants

**Figure 8:**
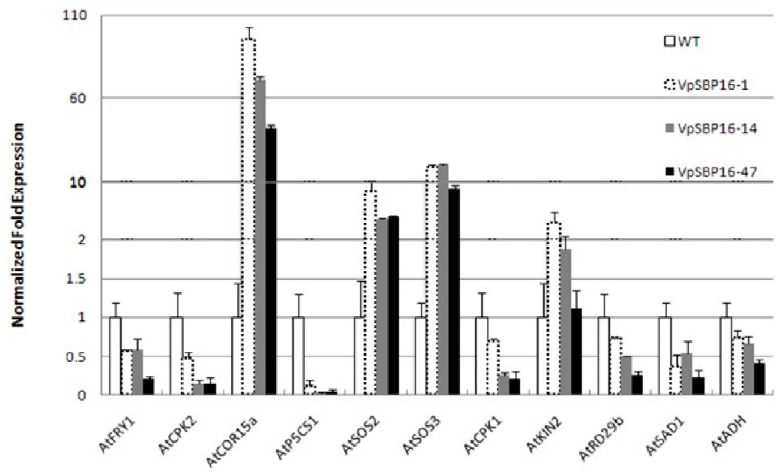
Expression levels of abiotic stress responsive genes in 3-week-old WT and *VpSBP16* transgenic *A. thaliana* plants. *AtActin1* was used as internal control for qRT-PCR. Mean values and SDs were obtained from three technical and three biological.

Drought and high salinity are common stress conditions that adversely affect plant growth and crop productivity. In order to better understand the mechanistic basis of the improved abiotic stress tolerance exhibited by the transgenic VpSBP16 plants, we measured the expression profiles of a number of abiotic stress-responsive genes (*AtSOS2*, *AtSOS3*, *AtFRY1*, *AtSAD1*, *AtADH*, *AtCOR15a*, *AtKIN2*, *AtP5CS1*, *AtRD29B*, *AtCDPK1* and *AtCDPK2*) in 3-week-old WT and transgenic plants using quantitative real-time PCR (qRT-PCR) analysis. We saw that the transgenic VpSBP16 overexpressing lines had higher expression levels of *AtSOS2*, *AtSOS3*, *AtCOR15a* and *AtKIN2*. In three transgenic lines VpSBP16-1, VpSBP16-14 and VpSBP16-47, the expression of *AtCOR15a* gene was 80, 65 and 35 times that of WT, respectively, while the expression of *AtSOS2* and *AtSOS3* was approximately 6-fold and 10-fold that of WT. The expression of *AtKIN2* gene in the transgenic lines was 2-fold greater than that of WT (Figure 8). However, the expression levels of *AtFRY1*, *AtSAD1*, *AtADH*, *AtP5CS1*, *AtRD29B*, *AtCDPK1* and *AtCDPK2* were lower than those in WT to varying degrees (Figure 8).

## 3. Discussion

Plants are exposed to a variety of biotic and abiotic stresses, such as cold, drought, salt stress and pathogens, during their life cycle, many of which can have an irreversible effect on growth and development. Many studies have identified transcription factors that are important for regulating plant responses to stress, including members of the WRKY, bZIP, AP2/ERF and MYB. In this regard, the functions of SBP family genes have been investigated in several plant species, but to date nothing has been reported regarding the role of SBP-box genes in grape responses to abiotic stress.

Members of the SBP-box gene family encode DNA binding proteins that function as transcription factors [26], and have been identified only in green plants, including taxa from single-cell algae to later-diverging land plants. Characteristics of a SBP domain are a ~74 amino acid (aa) residue region, a nuclear localization signal and the ability to bind DNA involving two zinc-finger domains [13]. In all cases, the SBP-domains are very similar, with a high sequence conservation at certain positions and the best-conserved aa residues are cysteines and histidines that are used to coordinate two zinc ions. Similarly well conserved are the basic amino acid residues that are thought to be involved in general and specific DNA binding, and in nuclear translocation of the proteins [9,13]. The VpSBP16 amino acid sequence contains all the above structural features, including the SBP-box and a putative nuclear localization signal (Figure 2). We confirmed that VpSBP16 is targeted to the nucleus and possesses transcriptional activation activity (Figure 3).

We studied the role of VpSBP16 in *A. thaliana* in the context of salinity and drought stress. Over-expression of *VpSBP16* in *A. thaliana* yielded transgenic lines with enhanced resistance to high osmoticum, dehydration, long-term drought, and salt stress compared with the WT.

The semi- and selective permeability of the plasma membrane are fundamentally important characteristics for maintaining normal plant physiological function. However, these permeability characteristics can be perturbed in response to stress, which can result in electrolyte leakage. We inferred the extent of the cellular injury, and by extension resistance to stress, by measuring electrolyte leakage. We found that the electrolyte leakage in WT was significantly higher than in the transgenic lines after the water loss treatment. The results were consistent with the improved tolerance to stress shown by the VpSBP16 transgenic lines.

Stress occurs when the root system cannot supply enough water to transpiring leaves to maintain a proper water balance. Accordingly, the ability of plant to overcome environmental stresses is influenced by its root system size and distribution. We found that the VpSBP16 transgenic lines had longer and a larger root system compared to the WT under the stresses, which likely helped in the tolerance of salt and drought stress by increasing water uptake. Under high saline condition, salt imposes an oxidative stress caused by the generation of ROS, such as singlet oxygen, H_2_O_2_ and O_2_^−^ [27]. Usually, this source of ROS does not result in serious harm to the plant, but under high salt and drought conditions, excess ROS is not eliminated rapidly enough, causing severe oxidative damage, and affecting physiology [28]. Therefore, the removal of excess ROS and the maintenance of ROS homeostasis are crucial for mitigating ROS toxicity and improving plant stress resistance.

Diverse transcription factors have been shown to contribute to the regulation of active oxygen scavenging. For example, overexpression of *OsMYB2* in rice was reported to reduce the content of ROS and enhance the drought tolerance [29], and under H_2_O_2_ stress, the expression of the banana WRKY family gene, *MusaWRKY71,* resulted in increased expression of peroxidase genes, which are part of the ROS scavenging system in banana seedlings [7]. Moreover, overexpression *JERF3* gene and *SlERF3* gene from *Solanum lycopersicum* in tobacco, and *TERF2* from *Solanum lycopersicum* and *SUB1A* gene from *Oryza sativa* in rice all improved the stress tolerance by enhancing the scavenging of ROS [30,31,32,33]. We observed that the levels of ROS in the *VpSBP16* transgenic lines were significantly lower than in WT plants, following exposure to the abiotic stresses.

We also examined the expression of some stress-responsive genes, such as *AtSOS2*, *AtSOS3*, *AtFRY1*, *AtSAD1*, *AtADH*, *AtCOR15*a, *AtKIN2*, *AtP5CS1*, *AtRD29B*, *AtCDPK1* and *AtCDPK2* to investigate the potential mechanisms of *VpSBP16* in stress responses. The stress-responsive gene candidates *COR15b*, *SOS2, SOS3*, exhibited a significantly higher expression level in the *VpSBP16* overexpression plants than in WT. Of these genes, *SOS3* was identified through genetic screening of salt-sensitive mutants and *SOS2* has been shown to function as a point of crosstalk between SOS and other signaling pathways [34]. Additionally, overexpression of *SlSOS2* (SlCIPK24) in tomato, *MdSOS2L1* in apple or *PtSOS2* genes (*PtSOS2.1*, *PtSOS2.2*, *PtSOS2.3*) in poplar was reported to confer salt tolerance to transgenic tomato, apple calli or poplar, respectively [35,36,37], further indicating its importance in abiotic stress resistance. However, the expression levels of AtP5CS1, AtCDPK1, AtCDPK2, AtRD29B, AtADH and AtFRY1 were down-regulated in the transgenic lines, contradicts previous research. The key enzyme in proline synthesis (P5CS1) is involved in the natural adaptation among Arabidopsis accessions in differential responses to osmotic stress [38,39]. Calcium-dependent protein kinases (CDPK) as the well-known Ca2+-sensor protein, play important roles in the plant response to salt stress [40]. The RD29B promoter region carrying several ABRE sequences and one DRE is controlled mainly by ABA and the -214 G-box in the AtAdh promoter also is the ABA response element [41,42]. AtRD29B, AtADH, AtFRY1 and AtSAD1 all confer both abiotic or biotic stress resistance in *A. thaliana* by involving in ABA signal transduction pathways [43,44,45,46]. Therefore, we speculate the resistance of the transgenic VpSBP16 plants against salt and drought stress have nothing to do with ABA signal transduction pathway and Ca^2+^-dependent signal network. However, further studies are necessary to unravel the reason of why these genes were down-regulated.

Based on these results we propose that overexpression of *VpSBP16* may increase resistance to salt and drought stress by regulating the SOS signaling cascade and ROS signaling. However, further studies will be needed to identify the exact molecular mechanisms by which the *VpSBP16* mediate responses to abiotic stresses. We noted that *AtCOR15*a was significantly induced in the *VpSBP16* overexpression plants, suggesting that *VpSBP16* may also be associated with cold stress responses. This will be a target of future research.

## 4. Materials and Methods

### 4.1. Plant Materials and Growth Conditions

Chinese wild grape, *Vitis pseudoreticulata* “Baihe-35-1”, used for cloning of *VpSBP16*, was grown in the grape repository of Northwest A&F University, Yangling, Shaanxi, China (34°20′ N, 108°24′ E). *A. thaliana* plants (T3 transgenic lines and wild-type (WT) ecotype Columbia-0) were grown at 22 °C, 70% relative humidity and long day (8 h dark, 16 h light) conditions. All experiments were repeated in triplicate and all samples were immediately frozen in liquid nitrogen and stored at −80 °C until further use.

### 4.2. *Isolation and Analysis of the* VpSBP16 cDNA

The cDNA was synthesized from total RNA samples extracted from *V. pseudoreticulata* ‘Baihe-35-1’ leaves. The experiments were performed essentially as previously described [47]. A pair of gene-specific primers (*VpSBP16*-F1 and *VpSBP16*-R1) (Table 1) were used to amplify the predicted *VpSBP16* ORF from the cDNA template with Taq DNA polymerase (TaKaRa Biotechnology, Dalian, China) and the following cycling program: 94 °C for 3 min, 35 cycles at 94 °C for 30 s, 58 °C for 30 s and 72 °C for 2 min; and extension at 72 °C for 10 min. The amplified products were cloned into the pGEM-Teasy vector (Promega, Madison, WI, USA) to generate pGEM-Teasy-*VpSBP16*, and transformed into *E. coli* strain *DH5α*. Positive clones were sequenced at TaKaRa Biotechnology. The sequences were analyzed to identify conserved regions using Conserved Domains (http://www.ncbi.nlm.nih.gov/Structure/cdd/wrpsb.cgi). The subcellular localization of *VpSBP16* was predicted using the Center for Biological Sequence analysis software (http://genome.cbs.dtu.dk/services/TargetP; http://genome.cbs.dtu. dk/services/SignalP/).

### 4.3. Subcellular Localization and Trans-Activation Assay

The *VpSBP16* CDS without the termination codon was amplified using *VpSBP16*-F2 and R2 with *Xba*I and *Kpn*I sites (Table 1) from the pGEM-Teasy-*VpSBP16* plasmid template, using *Taq* DNA polymerase (TaKaRa Biotechnology, Tokyo, Japan). The amplified product was inserted immediately upstream of the green fluorescent protein (GFP) coding sequence in the pBI221-GFP vector (Clontech Laboratories, Mountain View, CA, USA) and digested with the same restriction enzymes to generate pBI221-*VpSBP16*-GFP. Both the SBP16-containing vector and a background control vector with no insert were delivered into onion epidermal cells using a PDS-1000/He gene gun (Bio-Rad Laboratories Inc., Hercules, CA, USA) at 1100 psi as previously described [24], and then the cells were cultured on MS media in the dark at 22 °C for 18 h. Following cultivation, GFP signal was visualized using a Zeiss confocal microscope (LSM510; Carl Zeiss; Thornwood, NY, USA) with an excitation wavelength of 480 ± 20 nm and an emission wavelength of 510 ± 20 nm.

The coding regions of yeast *GAL4* and grape *VpSBP16* were separately ligated into the *Nco*I/*Bam*HI and *Xma*I/*Bam*HI sites of the GAL4 DNA-binding domain of the pGBKT7 vector (Clontech Laboratories), generating the plasmids pGBKT7-Gal4 (positive control) and pGBKT7-*VpSBP16*, respectively, using DNA fragments amplified with the gene-specific primers Gal4-F and R, and *VpSBP16* F3 and R3 (Table 1). The vectors were used in a trans-activation assay with a yeast assay system as described previously [47]. The resulting plasmids, pGBKT7-Gal4 and pGBKT7-VpSBP16, as well as the empty vector pGBKT7 (negative control), were transformed into yeast (*Saccharomyces cerevisiae*) AH109 cells, which were then streaked on SD/-Trp and SD/-Trp/-Ade/-His/X-α-Gal plates to observe yeast growth at 30 °C for 3–4 days.

### 4.4. Generation of Transgenic A. thaliana Plants Over-Expressing VpSBP16

The CDS of *VpSBP16* was amplified from the pGEM-Teasy-*VpSBP16* plasmid template using the gene-specific primers *VpSBP16*-F4 and R4 (Table 1), then inserted immediately downstream of the CaMV 35S promoter in the plant overexpression vector, pCambia2300 (Clontech Laboratories) to produce the plasmid pCambia2300-35S-*VpSBP16*. This construct was then introduced into *Agrobacterium tumefaciens* strain EHA105, which was in turn used to transform *A. thaliana* via the floral dip method [48]. Transgenic seeds (T1) were selected on MS agar medium supplemented with 60 mg L^−1^ kanamycin and T2 transgenic lines were selected on MS agar medium supplemented with 60 mg L^−1^ kanamycin and 150 mM NaCl. Three of the resulting T3 homozygous lines with most obvious phenotypes (SBP16-1, SBP16-14 and SBP16-47) and highest levels of expression were selected for further study.

### 4.5. Semi-Quantitative RT-PCR Analysis

Total *A. thaliana* RNA was extracted from entire WT and twelve transgene line (SBP16-1, SBP16-8, SBP16-14, SBP16-18, SBP16-23, SBP16-32, SBP16-35, SBP16-40, SBP16-47, SBP16-57, SBP16-61 and SBP16-67) plants using the E.Z.N.A.^®^ Plant RNA Kit (Omega Bio-tek, Norcross, GA, USA). Cycling parameters were as follows: 94 °C for 3 min, 25 cycles at 94 °C for 30 s, 60 °C for 30 s and 72 °C for 30 s, with a final elongation step at 72 °C for 10 min. An 8 μL sample of each PCR product was subsequently separated on a 1.2% (*w*/*v*) agarose gel, then stained with ethidium bromide and photographed under UV light. *Atactin1* (At2g37620) was amplified for use as an internal control. *VpSBP16*-F, *VpSBP16*-R and *AtActin1*-F, *AtActin1*-R specific primer sequences are listed in Table 1.

### 4.6. *Germination Assays*

For the seed germination assays, 100–150 seeds from each of three selected T3 homozygous lines and from WT plants were vernalized for 3 days at 4 °C, and then sown on MS medium or MS medium supplemented with 150 mM NaCl or 400 mM mannitol. The percentage of germinated seeds was calculated based on the number of seedlings that had reached the cotyledon stage at 2 weeks [49]. All seeds used for the germination analysis were harvested and stored at the same time under the same conditions. All germination assays were performed in triplicate.

### 4.7. *Salt and Drought Treatments* for Wild Type (WT) and *T3* Transgenic Lines

Seeds from WT and T3 transgenic lines were sterilized and vernalized for 3–4 days at 4 °C on the same MS medium, supplied with 150 mM NaCl or 400 mM mannitol, and then transferred to growth chambers. For the potted plants, three-week-old *A. thaliana* and transgenic lines were irrigated with 300 mM NaCl at 2-day intervals for 15 days or not watered, corresponding to salt and drought treatments, respectively. Plants that were well watered were used as the negative control. Following the drought treatment for 18 days, plants were re-watered for 3 days.

### 4.8. *Determination of the Water Loss Rate and* Electrolyte Leakage

For the determination of water loss, whole plants of 2-week-old transgenic and WT plants grown on the same MS medium were placed on dry filter paper and at 40–45% relative humidity at room temperature and weighed at the indicated times. The water loss rate was calculated based on the initial fresh weight of the samples and the experiment was repeated three times [50]. All plants were sampled after dehydration for 50 min and were vacuum-infiltrated with deionized water for 20 min. After 2 h, the conductivities (C1) of the solutions were determined using a conductivity detector. Subsequently, the seedlings were boiled for 20 min in deionized water and cooled to room temperature. The conductivities (C2) of the solutions was then determined. The C1 to C2 (C1/C2) ratios were calculated and used as a measure of the relative electrolyte leakage [51].

### 4.9. Detection of H_2_O_2_ and O_2_^−^

Excised leaves or whole plants were placed in 1 mg mL^−^^1^ diaminobenzidine (DAB) solution (Sigma, Steinheim, Germany) for 8 h to monitor H_2_O_2_ production, or were incubated in HEPES buffer (pH 7.5) containing 6 mM nitro blue tetrazolium(NBT) for 2 h to detect O_2_^−^ production. The samples were then cleared to remove chlorophyll at 80 °C in 80% (*v/v*) ethanol for 2 h and immersed in 10% (*v*/*v*) glycerol for observations [52,53].

### 4.10. Quantitative Real-Time RT-PCR Analysis

Total *A. thaliana* RNA was extracted from entire WT and three transgene line (SBP16-1, SBP16-14 and SBP16-69) plants using the E.Z.N.A.^®^ Plant RNA Kit (Omega Bio-tek, USA, R6827-01). First-strand cDNA for expression analysis was synthesized from 1 μg of DNase-treated total RNA using PrimeScript™ RTase (TaKaRa Biotechnology). Each reaction was done in triplicates with a reaction volume of 25 μL. Cycling parameters were 95 °C for 30 s, 40 cycles of 95 °C for 5 s, and 60 °C for 30 s. For dissociation curve analysis, a program including 95 °C for 15 s, followed by a constant increase from 60 °C to 95 °C, was included after the PCR cycles. Quantitative RT-PCR was conducted using SYBR green (Takara Biotechnology) with an IQ5 real time PCR machine (Bio-Rad, Hercules, CA, USA). Each reaction was performed in triplicate and data were analyzed as previously described [54]. The expression of stress-related genes such as *AtFRY1, AtSAD1, AtADH, AtP5CS1, AtRD29B, AtCDPK1* and *AtCDPK2* were measured by *RT-PCR* in WT and T3 transgenic lines. *Atactin1* (At2g37620) was amplified for use as an internal control. The sequences of the quantitative PCR primers are listed in Table 1.

## 5. Conclusions

Overexpression of *VpSBP16* may increase resistance to salt and drought stress by regulating the SOS signaling cascade and ROS signaling.

## Figures and Tables

**Table 1:** The information of primers used in this paper. F indicates forward primer, R indicates reversed primer and the underline indicates restriction sites.

Primer Pairs	Forward and Reverse Primers (5′-3′)	Restriction Enzyme Cutting Site
*VpSBP16*-F	F: GCCCAATTCGTCTGCAAGAAGT	none
*VpSBP16*-R	R: CACCACCCTTGCCACATGAAACA	none
*VpSBP16*-F1	F: ATGGGGTCTTGGAGCTACG	none
*VpSBP16*-R1	R: TTACGTTATCCGGAGGC	none
*AtSOS2*-F	F: ATTGAGGCTGTAGCGAAC	none
*AtSOS2*-R	R: GGTATTCCTTCTGTTGCC	none
*AtSOS3*-F	F: GGAGGAATCTCTTCGCTG	none
*AtSOS3*-R	R: CACGAAAGCCTTATCCACC	none
*AtFRY1*-F	F: CGCAGTAGCACTAGGATTG	none
*AtFRY1*-R	R: TTGACACCGAGTTTATTGG	none
*AtADH*-F	F: CTCTTGGTGCTGTTGGTTTAGG	none
*AtADH*-R	R: AATTGGCTTGTCATGGTCTTTC	none
*AtCOR15a*-F	F: GCGAACAATCCTTCACAG	none
*AtCOR15a*-R	R: CTTCGGGAGACCCACCT	none
*AtKIN2*-F	F: GTCAGAGACCAACAAGAATGCC	none
*AtKIN2*-R	R: TGACTCGAATCGCTACTTGTTC	none
*AtP5CS1*-F	F: TTCTCAGATGGTTTCCAGGTTG	none
*AtP5CS1*-R	R: TGGGAATGTCCTGATGGGTG	none
*AtRD29B*-F	F: GTGAAGATGACTATCTCGGTGGTC	none
*AtRD29B*-R	R: TACCAAGAGACTCAGCAATCTCTG	none
*AtCDPK1*-F	F: CCGTTTTGGGCTGAGACTGAA	none
*AtCDPK1*-R	R: CCATGGGTGAGCTAACACTTGC	none
*AtCDPK2*-F	F: GTCCATTACCTTCCCGGCATAT	none
*AtCDPK2*-R	R: CAGCAATTACCCGTAATGCCATT	none
*Atactin1*-F	F: AGGCACCTCTTAACCCTAAAGC	none
*Atactin1*-R	R: GGACAACGGAATCTCTCAGC	none
*VpSBP16*-F2	F: CGCTCTAGAATGGGGTCTTGGAGCTACG	*Xba*I site underlined
*VpSBP16*-R2	R: ATAGGTACCCGTTATCCGGAGGCAGAAAG	*Kpn*I site underlined
*VpSBP16*-F3	F: TATCCCGGGATGGGGTCTTGGAGCTACG	*Xma*I site underlined
*VpSBP16*-R3	R: GGCGGATCCTTACGTTATCCGGAGGCAGA	*BamH*I site underlined
*Gal4*-F	F: CCATGGTAATGAAGCTACTGTCTTCTAT	*Nco*I site underlined
*Gal4*-R	R: GGATCCTTACTCTTTTTTTGGGTTTG	*BamH*I site underlined
*VpSBP16*-F4	F: CACGGATCCATGGGGTCTTGGAGCTA	*BamH*I site underlined
*VpSBP16*-R4	R: GGCGGTACCTTACGTTATCCGGAGGC	*Kpn*I site underlined

## Supplementary Materials

Supplementary materials can be found at http://www.mdpi.com/1422-0067/19/4/940/s1.

## Abbreviations

CaMV	Cauliflower mosaic virus
ORF	Open reading frame
RT-PCR	Reverse transcription-polymerase chain reaction
MS	Murashige and Skoog
NBT	Nitro blue tetrazolium
DAB	Diaminobenzidine
ROS	Reactive oxygen species
H2O2	Hydrogen peroxide
O2^−^	Superoxide anion
SOS	Salt overly sensitive
SBP	Squamosa promoter binding protein
NLS	Nuclear localization signal
WT	Wild-type
