# Supplementary material for: Overexpression of a SBP-Box Gene (VpSBP16) from Chinese Wild Vitis Species in Arabidopsis Improves Salinity and Drought Stress Tolerance
Source: Int J Mol Sci. 2018 Mar 22;19(4):940. doi: 10.3390/ijms19040940 (PMC5979544; doi:10.3390/ijms19040940)
Supplement: Supplementary file 1 [file ijms-19-00940-s001.pdf]

Supplementary Files

Overexpression of a SBP-Box Gene (*VpSBP16*) from Chinese Wild *Vitis* Species in *Arabidopsis* Improves Salinity and Drought Stress Tolerance

Hongmin Hou [1,2,3,4], Hui Jia [1,2], Qin Yan [1] and Xiping Wang [1,2,*]

1 State Key Laboratory of Crop Stress Biology in Arid Areas, College of Horticulture, Northwest A&F University, Yangling, Shaanxi, 712100 , China; hmhou@qau.edu.cn (H.H.); jhhsunshine@nwafu.edu.cn (H.J.); yanqin0421@gmail.com (Q.Y.)

2 Key Laboratory of Horticultural Crop Biology and Germplasm Innovation in Northwest China, Ministry of Agriculture, Yangling, Shaanxi, 712100, China

3 College of Horticulture, Qingdao Agricultural University, Qingdao, Shandong, 266109, China

4 Qingdao Key Laboratory of Genetic Development and Breeding in Horticultural Plants, Qingdao Agricultural University, Qingdao, Shandong, 266109, China

* Correspondence: wangxiping@nwsuaf.edu.cn; Tel.: +86-298-708-2129

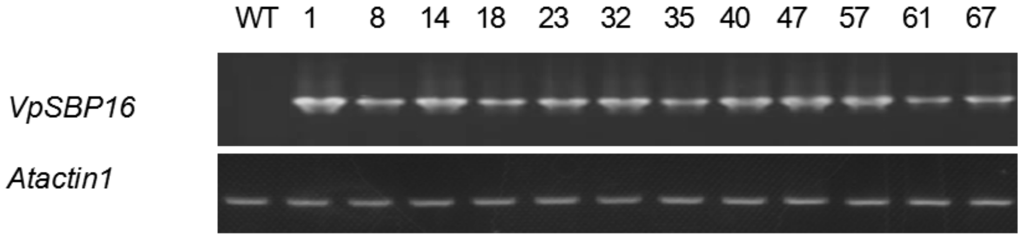

Figure S1. Semi-quantitative RT-PCR analysis of *VpSBP16* transcript levels in 3-week-old seeding leaves from WT and twelve transgenic lines (SBP16-1, SBP16-8 SBP16-14, SBP16-18, SBP16-23, SBP16-32, SBP16-35, SBP16-40, SBP16-47, SBP16-57, SBP16-61 and SBP16-67).
